# Low serum adiponectin level is associated with metabolic syndrome and is an independent marker of peripheral arterial stiffness in hypertensive patients

**DOI:** 10.1186/s13098-017-0247-8

**Published:** 2017-06-28

**Authors:** Ming-Chun Chen, Chung-Jen Lee, Chiu-Fen Yang, Yu-Chih Chen, Ji-Hung Wang, Bang-Gee Hsu

**Affiliations:** 1 Department of Pediatrics, Buddhist Tzu Chi General Hospital, Hualien, Taiwan; 2 Department of Nursing, Tzu Chi University of Science and Technology, Hualien, Taiwan; 3 Division of Cardiology, Buddhist Tzu Chi General Hospital, No. 707, Section 3, Chung-Yang Road, Hualien, 97002 Taiwan; 4 School of Medicine, Tzu Chi University, Hualien, Taiwan; 5 Division of Nephrology, Buddhist Tzu Chi General Hospital, No. 707, Section 3, Chung-Yang Road, Hualien, 97002 Taiwan

**Keywords:** Adiponectin, Peripheral arterial stiffness, Metabolic syndrome, Hypertension

## Abstract

**Background:**

Adiponectin has been implicated in metabolic syndrome (MetS) and arterial stiffness (AS). We aim to determine the relationship between serum adiponectin concentration as well as peripheral AS in hypertensive patients.

**Methods:**

Fasting blood samples were obtained from 101 hypertensive patients. Brachial-ankle pulse wave velocity (baPWV) was measured with an automatic pulse wave analyzer. Serum adiponectin concentrations were determined by using an enzyme immunoassay kit. A baPWV >14.0 m/s was defined as high AS.

**Results:**

MetS and high AS were present in 62.4 and 71.3% of the study population. Adiponectin was inversely associated with MetS and high AS (both *P* < 0.001). Serum higher high-density lipoprotein cholesterol (HDL-C) (*P* = 0.012), triglycerides (*P* = 0.001), C-reactive protein (*P* < 0.001), insulin (*P* = 0.027), body weight (*P* = 0.002), waist circumference (WC, *P* < 0.001), body mass index (*P* = 0.001) bilateral-baPWV (*P* < 0.001), systolic blood pressure (SBP, *P* < 0.001), diastolic blood pressure (DBP, *P* = 0.012), pulse pressure (*P* = 0.019), homeostasis model assessment of insulin resistance (HOMA1-IR (*P* = 0.026) and HOMA2-IR (*P* = 0.020)) and lower glomerular filtration rate (GFR, *P* = 0.029) were significantly associated with high AS. Multivariate logistic regression analysis of the factors significantly associated with AS revealed that adiponectin [odds ratio: 0.932, 95% confidence interval (CI) 0.881–0.985, *P* = 0.012], and SBP (odds ratio: 1.059, 95% CI 1.008–1.113, *P* = 0.022) were the independent predictors of arterial stiffness in hypertensive patients. Subgroup analysis revealed that SBP (odds ratio: 1.126, 95% CI 1.024–1.237, *P* = 0.014) and GFR (odds ratio: 0.858, 95% CI 0.739–0.996, *P* = 0.043) were the independent predictors of arterial stiffness in hypertensive patients without MetS; adiponectin (odds ratio: 0.909, 95% CI 0.931–0.996, *P* = 0.040) was the independent predictor of arterial stiffness in hypertensive patients with MetS.

**Conclusions:**

Hypoadiponectinemia has positive association with MetS and peripheral AS in hypertensive patients.

**Electronic supplementary material:**

The online version of this article (doi:10.1186/s13098-017-0247-8) contains supplementary material, which is available to authorized users.

## Background

Metabolic syndrome (MetS), a cluster of interrelated cardiometabolic risk factors, including visceral obesity, hypertension, hyperglycemia, elevated triglycerides (TG), and decreased high-density lipoprotein cholesterol (HDL-C) levels, is an independent risk factor for type 2 diabetes mellitus (DM) and cardiovascular (CV) disease [1]. The diabetes and heart disease risks are expected to rise by 2- to 5-fold over the next 5–10 years in patients with MetS compared with individuals without MetS [2].

Adiponectin secreted by adipocytes, contains a 247-amino acid protein with four differentiable domains [3]. Adiponectin plays an important role in obesity, insulin resistance (IR), MetS, and CV disease through its anti-inflammatory, anti-diabetic, and anti-atherogenic properties [4, 5].

Arterial stiffness, a pathological condition accompanying vascular damage, is one of the characteristics of CV disease. Pulse wave velocity (PWV), one of the widely used noninvasive methods to evaluate arterial stiffness, reflects segmental arterial elasticity in clinical practice [6]. Brachial-ankle pulse wave velocity (baPWV), which is more easily applied than carotid-femoral PWV, has been used as a surrogate of peripheral arterial stiffness and a marker for screening CV risk in the general population, in diabetes, and in hypertensive patients [6–9]. One meta-analysis revealed increased pooled relative risks for total CV events, CV mortality, and all-cause mortality for patients with high versus low baPWV [10].

MetS is associated with a higher prevalence of CV events and arterial stiffness [8]. A low serum adiponectin level is associated with both central elastic and peripheral muscular arterial stiffness in non-treated hypertensive patients [11, 12]. However, in the population of MetS patients, it remains uncertain whether decreased serum adiponectin is an independent risk factor for arterial stiffness. Thus, the present study provides further evidence of the relationship between decreased serum adiponectin and MetS. In addition, we investigated whether low serum adiponectin is independently associated with peripheral arterial stiffness evaluated by baPWV.

## Methods

### Patients

A total of 101 hypertensive participants were enrolled in this cross-sectional study conducted between January and December 2012 in the cardiovascular outpatient department at Buddhist Tzu Chi General Hospital, Hualien, Taiwan and the use of anti-hypertensive drugs for control blood pressure (BP) were according the Eighth Joint National Committee (JNC 8) guideline. By using standard mercury sphygmomanometers with appropriate cuff sizes, the BP of all patients was measured on the right arm by trained staff after sitting for at least 10 min in the morning. Systolic BP (SBP) and diastolic BP (DBP) were measured three times at 5-min intervals and were averaged for analysis. Hypertension was defined as SBP ≥140 mmHg, and/or DBP ≥90 mmHg, or having received any anti-hypertensive medication in the past 2 weeks among patients enrolled in this study. The Protection of Human Subjects Institutional Review Board of Tzu-Chi University and Hospital approved the study. All patients provided their informed consent before participation. Exclusion criteria included acute infection, acute myocardial infarction, and pulmonary edema at the time of blood sampling, or refusal to provide informed consent for the study.

### Anthropometric analysis

Body weight (BW) and height were measured with the participants in light clothing without shoes to the nearest 0.5 kg and half centimeter, respectively. Waist circumference (WC) was measured at the midpoint between the lowest ribs and the iliac crest with the hands on the hips. Body mass index (BMI) was calculated using the Quetelet’s formula as the weight in kilograms divided by the height in meters squared [2, 6, 13, 14].

### Biochemical investigations

After 8–12 h overnight fasting, blood samples (approximately 5 mL) of all participants were immediately centrifuged at 3000 *g* for 10 min. Serum levels of blood urea nitrogen (BUN), creatinine (Cre), fasting glucose, total cholesterol (TCH), TG, HDL-C, low-density lipoprotein cholesterol (LDL-C), total calcium, phosphorus, and C-reactive protein (CRP) were determined by using an autoanalyzer (COBAS Integra 800, Roche Diagnostics, Basel, Switzerland) [2, 6, 13, 14]. Serum adiponectin (SPI-BIO, Montigny le Bretonneux, France) and serum intact parathyroid hormone (iPTH) (Diagnostic Systems Laboratories, Texas, USA) concentrations were measured using a commercially available enzyme immunoassay and enzyme-linked immunosorbent assays, respectively [13]. The Modification of Diet in Renal Disease (MDRD) equation was used in this study for the calculation of estimated glomerular filtration rate (GFR).

### Metabolic syndrome and its components

The prevalence of MetS was determined by using the international diabetes federation definition [15], which includes central obesity with a waist circumference ≥90 cm (men) or ≥80 cm (women) (Chinese criteria) and meeting two or more of the following criteria: fasting serum glucose of ≥100 mg/dL, TG of ≥150 mg/dL, HDL-C level <40 mg/dL in men or <50 mg/dL in women or BP of ≥130/85 mmHg. Anti-hypertensive medication usage was considered as indicative of high BP in this analysis. Type 2 DM was defined using the World Health Organization criteria [16]. Individuals were classified as DM if the fasting plasma glucose was either 126 mg/dL or more or if the 2-h glucose during an oral glucose tolerance test was ≥200 mg/dL or if he/she was using diabetes medication (oral or insulin). Serum insulin levels were measured using the microparticle enzyme immunosorbent assay method by an autoanalyzer (Abbott Laboratories, Abbott Park, IL, USA). Insulin resistance was evaluated using a homeostasis model assessment of insulin resistance (HOMA-IR) as follows: HOMA1-IR = fasting plasma glucose (mg/dL) × fasting serum insulin (μU/mL)/405 [2, 17]; HOMA2-IR, is computer model which better reflects human physiology and is recalibrated to modern insulin assays (The HOMA2-IR model is available from http://www.OCDEM.ox.ac.uk) [18].

### Brachial-ankle pulse wave velocity measurements

BaPWV measurements were arranged immediately on the same day as blood sampling. After a 10-min rest in a quiet, temperature-controlled room, all participants were subjected to measurements in a supine position per the recommendations for user procedures in clinical applications of arterial stiffness. Heart rate and blood pressure (mean of three readings) were checked with an automatic upper-arm oscillometric device. The baPWV was measured in the right or left brachial artery to the ankle segments using an automatic pulse wave analyzer (VaSera VS-1000, Fukuda Denshi Co. Ltd., Tokyo, Japan) [6, 14]. In brief, cuffs were applied to the four extremities and electrocardiographic electrodes were attached to the upper arm. A microphone was placed on the sternal angle for phonocardiography. The subjects then rested in a supine position for 5 min. The baPWV was calculated by dividing the distance from the aortic valve to the ankle artery by the sum of the difference between the time the pulse waves were transmitted to the brachium and the time the same waves were transmitted to the ankle, and the time difference between the second heart sound on the phonocardiogram and the notch of the brachial pulse wave. To minimize cuff inflation effects on blood flow dynamics, pulse waves were measured with the cuffs inflated to less than the DBP (50 mmHg). In this study, left or right baPWV values of >14.0 m/s were used to define the high arterial stiffness group [6].

### Statistical analysis

All statistical analyzes were performed using the statistical package for the social sciences (SPSS) version 19.0 (SPSS Inc., Chicago, IL, USA). The distribution pattern of the variables was checked using the Kolmogorov–Smirnov test. Normally distributed variables are expressed as mean ± standard deviation and comparisons between patients were performed using the Student’s independent *t* test (two-tailed). Data not normally distributed are expressed as medians and interquartile ranges, and comparisons between patients were performed by using the Mann–Whitney U test (TG, fasting glucose, CRP, iPTH, insulin, HOMA1-IR, HOMA2-IR, and adiponectin). Data expressed as the number of patients were analyzed by the χ^2^ test. Because TG, fasting glucose, CRP, iPTH, insulin, HOMA1-IR, HOMA2-IR, and adiponectin were not normally distributed, they underwent base 10 logarithmic transformations to achieve normality. Variables that were significantly associated with arterial stiffness in hypertensive patients or hypertensive patients without MS or hypertensive patients with MS were tested for independence by multivariate logistic regression analysis. A *P* value <0.05 was considered statistically significant.

## Results

Demographic, clinical, and biochemical characteristics of the 101 hypertensive patients with or without MetS are presented in Table 1. A total of 44 patients (43.6%) had DM and 67 patients (66.3%) had a medical history of CAD. Sixty-three patients (62.4%) with MetS had lower serum adiponectin (*P* < 0.001) and HDL-C (*P* = 0.002) levels, higher serum TG (*P* < 0.001), fasting glucose (*P* < 0.001), CRP (*P* < 0.001), insulin (*P* = 0.004) levels, elevated BW (*P* < 0.001), WC (*P* < 0.001), BMI (*P* < 0.001), left and right-baPWV (*P* < 0.001), SBP (*P* = 0.014), HOMA1-IR (*P* = 0.001), HOMA2-IR (*P* = 0.002) values, and higher percentages of type 2 DM (*P* = 0.039) than those in the non-MetS group.

**Table 1:** Clinical variables of the 101 hypertensive patients with or without metabolic syndrome

Variables	All participants (n = 101)	No metabolic syndrome (n = 38)	Metabolic syndrome (n = 63)	*P* value
Age (years)	64.88 ± 9.58	66.13 ± 10.14	64.13 ± 9.23	0.311
Height (cm)	161.27 ± 8.36	162.22 ± 7.24	160.70 ± 8.98	0.377
Body weight (kg)	69.73 ± 13.01	63.34 ± 10.80	73.59 ± 12.78	<0.001*
Waist circumference (cm)	93.37 ± 11.55	84.82 ± 9.44	98.52 ± 9.50	<0.001*
Body mass index (kg/m^2^)	26.71 ± 3.87	23.99 ± 3.24	28.34 ± 3.27	<0.001*
Left baPWV (m/s)	15.22 ± 3.45	12.65 ± 3.06	16.77 ± 2.67	<0.001*
Right baPWV (m/s)	15.32 ± 3.69	12.46 ± 3.01	17.04 ± 2.91	<0.001*
Systolic blood pressure (mmHg)	133.44 ± 16.68	128.21 ± 13.70	136.59 ± 17.60	0.014*
Diastolic blood pressure (mmHg)	74.52 ± 10.73	72.34 ± 10.64	75.84 ± 10.65	0.113
Pulse pressure (mmHg)	58.91 ± 14.92	55.87 ± 12.71	60.75 ± 15.92	0.112
Total cholesterol (mg/dL)	174.10 ± 40.81	176.95 ± 39.80	172.38 ± 41.62	0.588
Triglycerides (mg/dL)	127.00 (91.00–177.00)	107.00 (73.75–128.50)	150.00 (104.00–218.00)	<0.001*
HDL-C (mg/dL)	45.06 ± 13.07	50.08 ± 14.25	42.03 ± 11.39	0.002*
LDL-C (mg/dL)	102.29 ± 31.55	105.16 ± 31.71	100.56 ± 31.58	0.480
Fasting glucose (mg/dL)	108.00 (96.00–145.50)	97.50 (89.75–115.25)	122.00 (103.00–161.00)	<0.001*
Blood urea nitrogen (mg/dL)	17.38 ± 5.73	16.13 ± 3.97	18.13 ± 6.48	0.090
Creatinine (mg/dL)	1.13 ± 0.32	1.09 ± 0.28	1.15 ± 0.35	0.318
Glomerular filtration rate (mL/min)	68.93 ± 19.98	72.82 ± 19.55	66.59 ± 20.02	0.130
Total calcium (mg/dL)	9.14 ± 0.38	9.03 ± 0.38	9.20 ± 0.36	0.035*
Phosphorus (mg/dL)	3.51 ± 0.52	3.40 ± 0.46	3.58 ± 0.55	0.095
Calcium-phosphorous product (mg^2^/dL^2^)	32.09 ± 5.25	30.71 ± 4.66	32.92 ± 5.45	0.041*
Intact parathyroid hormone (pg/mL)	46.35 (32.40–61.90)	49.60 (34.25–67.58)	44.15 (28.88–57.55)	0.009*
C-reactive protein (mg/dL)	0.20 (0.14–0.27)	0.16 (0.12–0.19)	0.24 (0.17–0.36)	<0.001*
Insulin (uIU/mL)	11.99 (7.98–25.16)	9.86 (6.06–15.14)	15.22 (9.22–28.99)	0.004*
HOMA1-IR	3.57 (2.27–7.45)	2.37 (1.50–4.58)	4.35 (2.93–8.72)	0.001*
HOMA2-IR	1.63 (1.10–3.30)	1.32 (0.81–1.97)	2.14 (1.33–3.91)	0.002*
Adiponectin (μg/mL)	28.40 (23.09–39.90)	36.92 (26.53–47.17)	25.55 (21.00–33.66)	<0.001*
Female (%)	34 (33.7)	9 (23.7)	25 (39.7)	0.099
Diabetes (%)	44 (43.6)	9 (23.7)	35 (55.6)	0.002*

Values for continuous variables given as mean ± standard deviation and compared by Student’s *t* test; variables not normally distributed given as medians and interquartile range and compared by Mann–Whitney U test; values are presented as number (%), and analysis was performed using the Chi square test

*baPWV* brachial-ankle pulse wave velocity, *HDL-C* high-density lipoprotein cholesterol, *LDL-C* low-density lipoprotein cholesterol, *HOMA-IR* homeostasis model assessment of insulin resistance

* *P* < 0.05 was considered statistically significant

The demographics, clinical characteristics, biochemical data, and comorbidities of our 101 hypertensive patients with or without peripheral arterial stiffness are presented in Table 2. Seventy-two patients (71.3%) with high arterial stiffness had lower serum adiponectin (*P* < 0.001) and HDL-C (*P* = 0.012) levels, higher serum TG (*P* = 0.001), CRP (*P* < 0.001), insulin (*P* = 0.027) levels, elevated BW (*P* = 0.002), WC (*P* < 0.001), BMI (*P* = 0.001), left and right-baPWV (*P* < 0.001), SBP (*P* < 0.001), DBP (*P* = 0.012), pulse pressure (*P* = 0.019), HOMA1-IR (*P* = 0.026), HOMA2-IR (*P* = 0.020) values and lower GFR (*P* = 0.029) than those in the low arterial stiffness group. Uni- and multivariate linear analyses of the clinical variables associated with left and right baPWV levels in hypertensive patients are shown in Additional file 1: Table S1 and Table S2.

**Table 2:** Clinical variables of the 101 hypertensive patients with or without arterial stiffness

Variables	Low arterial stiffness group (n = 29)	High arterial stiffness group (n = 72)	*P* value
Age (years)	64.00 ± 8.90	65.24 ± 9.88	0.560
Height (cm)	160.21 ± 6.80	161.70 ± 8.93	0.419
Body weight (kg)	63.59 ± 10.28	72.21 ± 13.23	0.002*
Waist circumference (cm)	86.03 ± 10.36	96.32 ± 10.72	<0.001*
Body mass index (kg/m^2^)	24.80 ± 3.81	27.48 ± 3.65	0.001*
Left baPWV (m/s)	11.24 ± 2.40	16.82 ± 2.32	<0.001*
Right baPWV (m/s)	11.15 ± 2.48	16.99 ± 2.60	<0.001*
Systolic blood pressure (mmHg)	123.79 ± 14.84	137.32 ± 15.87	<0.001*
Diastolic blood pressure (mmHg)	70.34 ± 9.08	76.21 ± 10.93	0.012*
Pulse pressure (mmHg)	53.45 ± 13.83	61.11 ± 14.86	0.019*
Total cholesterol (mg/dL)	178.93 ± 40.20	172.15 ± 41.17	0.453
Triglycerides (mg/dL)	105.00 (63.50–130.00)	143.00 (101.75–208.50)	0.001*
HDL-C (mg/dL)	50.17 ± 11.71	43.00 ± 13.10	0.012*
LDL-C (mg/dL)	106.62 ± 30.51	100.54 ± 32.00	0.384
Fasting glucose (mg/dL)	99.00 (93.00–123.50)	113.00 (98.00–158.50)	0.066
Blood urea nitrogen (mg/dL)	16.72 ± 5.94	17.64 ± 5.67	0.471
Creatinine (mg/dL)	1.05 ± 0.33	1.16 ± 0.32	0.132
Glomerular filtration rate (mL/min)	75.76 ± 20.76	66.18 ± 19.11	0.029*
Total calcium (mg/dL)	9.07 ± 0.36	9.16 ± 0.39	0.286
Phosphorus (mg/dL)	3.46 ± 0.51	3.53 ± 0.53	0.531
Calcium-phosphorous product (mg^2^/dL^2^)	31.40 ± 5.05	32.37 ± 5.34	0.405
Intact parathyroid hormone (pg/mL)	51.10 (36.15–69.05)	44.40 (29.00–58.60)	0.224
C-reactive protein (mg/dL)	0.16 (0.12–0.20)	0.23 (0.16–0.32)	<0.001*
Insulin (uIU/mL)	9.61 (5.52–18.66)	13.79 (9.10–27.04)	0.027*
HOMA1-IR	2.76 (1.62–4.70)	3.89 (2.59–8.55)	0.026*
HOMA2-IR	1.30 (0.78–2.38)	1.96 (1.27–3.70)	0.020*
Adiponectin (μg/mL)	39.91 (27.06–52.88)	26.89 (21.15–34.41)	<0.001*

Values for continuous variables given as mean ± standard deviation and compared by Student’s *t* test; variables not normally distributed given as medians and interquartile range and compared by Mann–Whitney U test

*baPWV* brachial-ankle pulse wave velocity, *HDL-C* high-density lipoprotein cholesterol, *LDL-C* low-density lipoprotein cholesterol, *iPTH* intact parathyroid hormone, *HOMA-IR* homeostasis model assessment of insulin resistance

* *P* < 0.05 was considered statistically significant

The drugs used included angiotensin-converting enzyme inhibitors (ACEi; n = 37; 36.6%), angiotensin receptor blockers (ARB; n = 56; 55.4%), β-blockers (n = 56; 55.4%), calcium channel blockers (CCB; n = 44; 43.6%), thiazides (n = 11; 10.9%), statins (n = 52; 51.2%), and fibrates (n = 11; 10.9%). Peripheral arterial stiffness did not differ statistically by sex, CAD, or use of ACEi, ARB, β-blockers, CCB, thiazides, statins, or fibrates, but there were statistically significant differences in diabetes and MetS (both *P* < 0.001) among hypertensive patients (Table 3).

**Table 3:** Baseline characteristics of the 101 hypertensive patients with or without arterial stiffness

Characteristic	Low arterial stiffness group (%)	High arterial stiffness group (%)	*P* value
Gender			
Male	19 (65.5)	48 (66.7)	0.912
Female	10 (34.5)	24 (33.3)	
Diabetes			
No	21 (72.4)	36 (50.0)	<0.001*
Yes	8 (27.6)	36 (50.0)	
Metabolic syndrome			
No	22 (75.9)	16 (22.2)	<0.001*
Yes	7 (24.1)	56 (77.8)	
ACE inhibitor use			
No	17 (58.6)	47 (65.3)	0.530
Yes	12 (41.4)	25 (34.7)	
ARB use			
No	14 (48.3)	31 (43.1)	0.633
Yes	15 (51.7)	41 (56.9)	
β-blocker use			
No	12 (41.4)	33 (45.8)	0.684
Yes	17 (58.6)	39 (54.2)	
CCB use			
No	15 (51.7)	42 (58.3)	0.544
Yes	14 (48.3)	30 (41.7)	
Thiazide use			
No	23 (78.3)	57 (79.2)	0.987
Yes	6 (20.7)	5 (20.8)	
Statin			
No	12 (41.4)	31 (43.1)	0.877
Yes	17 (58.6)	35 (56.9)	
Fibrate			
No	23 (79.3)	64 (88.9)	0.208
Yes	6 (20.7)	5 (11.3)	

Data are expressed as number of patients and analysis was performed by using the Chi square test

*ARB* angiotensin-receptor blocker, *ACE* angiotensin-converting enzyme, *CCB* calcium-channel blocker

* *P* < 0.05 was considered statistically significant

Subgroup analysis of arterial stiffness in hypertensive patients with or without MetS is presented in Table 4. High arterial stiffness patients without MetS had advanced age (*P* = 0.023), higher bilateral baPWV (both *P* < 0.001), and SBP (*P* < 0.001) values, and lower GFR (*P* = 0.016) than those in the low arterial stiffness group. Hypertensive patients with MetS and baPWV values >14 m/sec had higher triglyceride (*P* < 0.026) and CRP (*P* = 0.015) levels, and lower HDL-C (*P* = 0.003) and adiponectin (*P* < 0.001) levels than hypertensive patients with MetS with baPWV values ≤14 m/sec.

**Table 4:** Clinical variables with arterial stiffness in the hypertensive patients with or without metabolic syndrome

Variables	No metabolic syndrome (n = 38)			Metabolic syndrome (n = 63)		
	Low AS group (n = 22)	High AS group (n = 16)	*P* value	Low AS group (n = 7)	High AS group (n = 56)	*P* value
Age (years)	63.00 ± 8.94	70.44 ± 10.37	0.023*	67.14 ± 8.65	63.75 ± 9.30	0.363
Height (cm)	161.237 ± 5.95	163.59 ± 8.73	0.326	157.00 ± 8.72	161.16 ± 8.98	0.251
Body weight (kg)	62.36 ± 11.20	64.69 ± 10.42	0.520	67.43 ± 5.65	74.36 ± 13.23	0.178
Waist circumference (cm)	84.09 ± 11.04	85.81 ± 6.88	0.586	92.14 ± 4.18	99.32 ± 9.70	0.646
Body mass index (kg/m^2^)	23.95 ± 3.77	24.05 ± 2.44	0.930	27.46 ± 2.67	28.45 ± 3.34	0.454
Left baPWV (m/s)	10.64 ± 2.41	15.43 ± 0.86	<0.001*	13.13 ± 0.98	17.22 ± 2.45	<0.001*
Right baPWV (m/s)	10.56 ± 2.52	15.06 ± 1.03	<0.001*	13.00 ± 1.13	17.55 ± 2.66	<0.001*
Systolic blood pressure (mmHg)	122.50 ± 13.87	136.06 ± 8.97	0.002*	127.86 ± 18.14	137.68 ± 17.39	0.166
Diastolic blood pressure (mmHg)	69.50 ± 9.53	76.25 ± 11.14	0.052	73.00 ± 7.53	76.20 ± 10.97	0.458
Pulse pressure (mmHg)	53.00 ± 12.95	59.81 ± 11.62	0.104	54.86 ± 17.40	61.48 ± 15.74	0.303
Total cholesterol (mg/dL)	178.14 ± 37.05	175.31 ± 44.50	0.832	181.43 ± 52.21	171.25 ± 40.55	0.546
Triglycerides (mg/dL)	107.00 (72.25–128.50)	101.00 (76.25–138.00)	0.429	65.00 (45.00–164.00)	153.00 (114.75–220.25)	0.026*
HDL-C (mg/dL)	49.00 ± 12.39	51.56 ± 16.78	0.591	53.86 ± 9.01	40.55 ± 10.83	0.003*
LDL-C (mg/dL)	107.41 ± 28.40	102.06 ± 36.51	0.614	104.14 ± 38.86	100.11 ± 30.95	0.753
Fasting glucose (mg/dL)	98.00 (92.50–121.25)	95.50 (89.00–106.00)	0.636	113.00 (96.00–124.00)	123.50 (104.25–167.00)	0.344
Blood urea nitrogen (mg/dL)	16.00 ± 3.60	16.31 ± 4.56	0.815	19.00 ± 10.55	18.02 ± 5.93	0.709
Creatinine (mg/dL)	1.01 ± 0.27	1.19 ± 0.28	0.059	1.17 ± 0.49	1.15 ± 0.33	0.890
Glomerular filtration rate (mL/min)	79.23 ± 18.66	64.00 ± 17.66	0.016*	64.86 ± 24.69	66.80 ± 19.61	0.811
Total calcium (mg/dL)	9.03 ± 0.31	9.08 ± 0.48	0.864	9.22 ± 0.46	9.20 ± 0.35	0.850
Phosphorus (mg/dL)	3.39 ± 0.53	3.40 ± 0.37	0.953	3.66 ± 0.40	3.56 ± 0.57	0.677
Ca X IP product (mg^2^/dL^2^)	30.64 ± 5.07	30.81 ± 4.17	0.912	33.77 ± 4.49	32.81 ± 5.59	0.663
iPTH (pg/mL)	49.20 (32.78–64.83)	50.35 (36.13–74.60)	0.881	56.80 (37.90–75.90)	43.70 (27.00–54.40)	0.389
C-reactive protein (mg/dL)	0.16 (0.12–0.19)	0.17 (0.12–0.20)	0.581	0.17 (0.12–0.20)	0.25 (0.18–0.38)	0.015*
Insulin (uIU/mL)	9.68 (5.64–19.54)	10.11 (6.26–13.67)	0.662	9.61 (5.05–17.91)	16.75 (9.56–29.32)	0.155
HOMA1-IR	2.56 (1.59–5.01)	2.34 (1.41–3.80)	0.791	3.34 (1.53–4.67)	4.77 (2.98–9.22)	0.165
HOMA2-IR	1.27 (0.79–2.48)	1.35 (0.81–1.81)	0.919	1.48 (0.71–2.31)	2.17 (1.36–3.91)	0.114
Adiponectin (μg/mL)	38.98 (28.29–50.67)	35.29 (25.62–44.77)	0.272	44.71 (24.14–61.15)	25.41 (20.66–32.69)	<0.001*

Values for continuous variables given as mean ± standard deviation and compared by Student’s *t* test; variables not normally distributed given as medians and interquartile range and compared by Mann–Whitney U test

*AS* arterial stiffness, *baPWV* brachial-ankle pulse wave velocity, *HDL-C* high-density lipoprotein cholesterol, *LDL-C* low-density lipoprotein cholesterol, *iPTH* intact parathyroid hormone, *Ca X IP product* Calcium-phosphorous product *HOMA-IR* homeostasis model assessment of insulin resistance

* *P* < 0.05 was considered statistically significant

Multivariate logistic regression analysis of the factors (diabetes, age, BW, WC, BMI, SBP, DBP, pulse pressure, triglyceride, HDL-C, GFR, CRP, insulin, HOMA1-IR, HOMA2-IR, and adiponectin) significantly associated with arterial stiffness revealed that adiponectin (odds ratio: 0.932, 95% confidence interval (CI) 0.881–0.985, *P* = 0.012), and SBP (odds ratio: 1.059, 95% CI 1.008–1.113, *P* = 0.022) were the independent predictors of arterial stiffness in hypertensive patients. Subgroup analysis revealed that SBP (odds ratio: 1.126, 95% CI 1.024–1.237, *P* = 0.014) and GFR (odds ratio: 0.858, 95% CI 0.739–0.996, *P* = 0.043) were the independent predictors of arterial stiffness in hypertensive patients without MetS, while adiponectin (odds ratio: 0.909, 95% CI 0.931–0.996, *P* = 0.040) was the independent predictor of arterial stiffness in hypertensive patients with MetS (Table 5).

**Table 5:** Multivariate logistic regression analysis to determine factors correlated to arterial stiffness

Variables	Hypertensive patients (n = 101)			Hypertension without MS (n = 38)			Hypertension with MS (n = 63)		
	OR	95% CI	*P* value	OR	95% CI	*P* value	OR	95% CI	*P* value
Adiponectin (μg/mL)	0.932	0.881–0.985	0.012*	–	–	–	0.909	0.931–0.996	0.040*
SBP (mmHg)	1.059	1.008–1.113	0.022*	1.126	1.024–1.237	0.014*	–	–	–
GFR (mL/min)	–	–	–	0.858	0.739–0.996	0.043*	–	–	–

Multivariate logistic regression analysis of diabetes, age, waist circumference, body weight, body mass index, systolic blood pressure, diastolic blood pressure, pulse pressure, triglyceride, HDL-C, glomerular filtration rate, CRP, insulin, HOMA1-IR, HOMA2-IR, and adiponectin

*OR* odds ratio, *CI* confidence interval, *SBP* systolic blood pressure, *HDL-C* high density lipoprotein cholesterol, *CRP* C-reactive protein, *HOMA-IR* homeostasis model assessment of insulin resistance

* *P* < 0.05 was considered statistically significant

## Discussion

Serum adiponectin levels were significantly lower in hypertensive patients with MetS and had association with arterial stiffness in hypertensive patients. Decreased serum adiponectin level is associated with the development of arterial stiffness in hypertensive individuals with MetS, but this association lost significance in the group without MetS.

Insulin resistance with compensatory hyperinsulinemia plays a central role in MetS [2]. Insulin metabolism is interrelated with adiponectin secretion and action [19]. In recent years, adiponectin has been recognized as an insulin sensitizer involved in energy metabolism and regulation of many biological processes, such as migration, proliferation, apoptosis, and inflammation [20]. Epidemiological studies found that decreased serum total and high-molecular-weight adiponectin were associated with MetS in 546 Japanese-Americans and in 750 Japanese during a 3–4-year follow up [21, 22]. Another study with 2044 participants showed that hypoadiponectinemia was significantly associated with incident MetS, even after adjusting for BMI, CRP, and HOMA-IR [23]. Recently, Lindberg et al. even reported that hypoadiponectinemia at baseline and during a 9-year follow up had significant positive correlations with incident MetS independently of gender, age, and BMI as well as glucose, TG, HDL-C levels, and GFR [4]. Our study also revealed that lower adiponectin, elevated insulin and HOMA-IR values, higher CRP, fasting glucose, TG, and lower HDL-C levels and increased BW, WC, BMI, SBP, and bilateral baPWV values exhibited a statistically significant difference between patients with hypertensive MetS and those without MetS, suggesting that circulating adiponectin concentration has association with insulin resistance and other metabolic risks.

Arterial stiffness, which is associated with low-grade inflammation and vascular element reorganization with unregulated collagen and elastin fiber, can be assessed simply and noninvasively by measuring PWV [24]. Many studies have demonstrated that PWV is a strong independent prognostic factor of CV disease as well as mortality [7–10]. Epidemiological studies revealed that SBP, TG, and HDL-C are independent predictors of baPWV in 835 young adults and MetS and its components (fasting glucose and BP) had an independent association with the incidence or future progression of arterial stiffness among 1518 community-dwelling Taiwanese adults aged over 40 years [25]. Other studies also stated that age, BMI, BP, prevalence of antihypertensive medication, and renal function were significantly associated with arterial stiffness, and increased baPWV values were noted while these risk factors accumulated [26–28]. In addition, stiffness of large conduit arteries causes elevated SBP and decreased DBP, resulting in increasing pulse pressure and PWV, a vicious circle for developing future CV events [29]. Moreover, old age as well as MetS components may accelerate arterial stiffness via activating extracellular matrix metalloproteinases, a major determinant of vascular remodeling and arterial stiffness [30, 31]. Our study also revealed that lower adiponectin levels, accompanied by traditional risk factors, such as lower GFR, elevated insulin and HOMA-IR values, higher CRP and TG and lower HDL-C levels, and increased BW, WC, BMI, SBP, DBP, pulse pressure, and bilateral baPWV values, demonstrated statistically significant differences between patients with hypertensive high arterial stiffness and those with low arterial stiffness. Moreover, patients with high arterial stiffness had significantly higher rate of diabetes and MetS.

Hypoadiponectinemia is involved in the pathophysiology of atherosclerosis. In a study of 445 Chinese participants aged ≥40 years, circulating adiponectin levels were independently associated with baPWV in a community-based population after adjustment for gender, age, BMI, renal clearance, and number of MetS components [32]. In line with our findings, a report by Kawamoto et al. revealed that lower serum high-molecular-weight adiponectin was significantly associated with elevated mean PWV as well as BMI, SBP, DBP, and GFR after multiple linear regressions analyzes for mean PWV [25]. Besides, circulating high-molecular-weight adiponectin levels also had a dose-dependent association with mean PWV and exerted greater influence than alterations in BP levels over arterial stiffness mechanics [25]. Furthermore, hypoadiponectinemia is a marker of oxidized low-density lipoproteins (oxLDL), inducing extensive endothelial dysfunction [33]. In the presence of inflammatory and oxidative vascular injury, hypoadiponectinemia causes vascular dysfunction and enhances PWV elevation [12].

Subgroup analysis of our data showed that bilateral baPWV values were significantly elevated in the high arterial stiffness group among hypertensive patients with or without MetS. In addition, serum adiponectin, CRP, TG, and HDL-C levels were significantly associated with high arterial stiffness than lower arterial stiffness in MetS patients, but this association was not prominent in the non-MetS group. Furthermore, MetS patients with high arterial stiffness revealed even further increased left and right baPWV values and CRP levels as well as decreased serum adiponectin levels than non-MetS patients with high arterial stiffness, indicating that these metabolic profiles had a more prominent influence on arterial stiffness in the MetS population. Multivariable analysis of the determining factors correlated with arterial stiffness in our study also confirmed that hypoadiponectinemia is a key independent predictor of arterial stiffness among hypertensive patients. When we take MetS into consideration, a lower serum adiponectin level is still a strong risk factor for the development of arterial stiffness. However, adiponectin level was not an independent factor, and only SBP and GFR are crucial independent predictors of arterial stiffness among hypertensive patients without MetS. Our findings are consistent with previous study reported by Chen et al. that baPWV was significantly increased in a south Chinese population with MetS relative to those without MetS [34].

There are some limitations to the current study. Firstly, this was a cross-sectional study and therefore, further longitudinal studies are needed before a cause-effect relationship between serum adiponectin and arterial stiffness can be established in the hypertensive population. Secondly, adiponectin has three different oligomeric forms in the circulation: low-molecular weight trimers, medium-molecular weight hexamers, and high-molecular-weight multimers of 12–18 subunits. Many studies have stated that high-molecular-weight adiponectin is the most active and powerful adiponectin, a strong association between serum total and high-molecular-weight adiponectin was reported [21, 35]. In addition, both circulating total and high-molecular-weight adiponectin levels have been inversely associated with parameters of insulin resistance, endothelial dysfunction, and inflammation, as well as with MetS prevalence or incidence, indicating that the use of total adiponectin concentration is a well-accepted surrogate of MetS and arterial stiffness evaluation [4, 21]. Moreover, the baPWV value is largely dependent on current SBP and careful attention must be paid when evaluates peripheral arterial stiffness by using the baPWV [36]. Therefore peripheral arterial stiffness evaluated by baPWV value may be overestimated in hypertensive patients. Further studies are required to elucidate the relationship between baPWV value and adiponectin levels in hypertensive patients.

## Conclusions

Adiponectin is recognized as a consistent and significant parameter associated with MetS. Taken together, our results indicate that hypoadiponectinemia is positively associated with peripheral AS in hypertensive patients. Further prospective studies are needed to confirm the mechanisms underlying this association.

## Additional file

**Additional file 1: Table S1.** Correlation between left brachial-ankle pulse wave velocity levels and clinical variables among 101 hemodialysis patients. **Table S2.** Correlation between right brachial-ankle pulse wave velocity levels and clinical variables among 101 hemodialysis patients.

## Abbreviations

MetS: metabolic syndrome
AS: arterial stiffness
baPWV: brachial-ankle pulse wave velocity
HDL-C: high-density lipoprotein cholesterol
TG: triglycerides
DM: diabetes mellitus
CV: cardiovascular
IR: insulin resistance
PWV: pulse wave velocity
BP: blood pressure
SBP: systolic blood pressure
DBP: diastolic blood pressure
CAD: coronary artery disease
BW: body weight
WC: waist circumference
BMI: body mass index
BNU: blood urea nitrogen
Cre: creatinine
TCH: total cholesterol
LDL-C: low-density lipoprotein cholesterol
CRP: C-reactive protein
iPTH: intact parathyroid hormone
MDRD: the modification of diet in renal disease
GFR: glomerular filtration rate
HOMA-IR: homeostasis model assessment of insulin resistance
ACEi: angiotensin-converting enzyme inhibitors
ARB: angiotensin receptor blockers
CCB: calcium channel blockers
PPAR-γ: peroxisome proliferator-activated receptor-γ
