# Supplementary material for: Low serum adiponectin level is associated with metabolic syndrome and is an independent marker of peripheral arterial stiffness in hypertensive patients
Source: Diabetol Metab Syndr. 2017 Jun 28;9:49. doi: 10.1186/s13098-017-0247-8 (PMC5490214; doi:10.1186/s13098-017-0247-8)
Supplement: Supplementary file 1 — Additional file 1: Table S1. Correlation between left brachial-ankle pulse wave velocity levels and clinical variables among 101 hemodialysis patients. Table S2. Correlation between right brachial-ankle pulse wave velocity levels and clinical variables among 101 hemodialysis patients. [file 13098_2017_247_MOESM1_ESM.doc]

Table S1. Correlation between left brachial-ankle pulse wave velocity levels and clinical variables among 101 hemodialysis patients

| **Variables** | **Left brachial-ankle pulse wave velocity (m/s)** | | | | | |
| --- | --- | --- | --- | --- | --- | --- |
|  | **Univariate** | |  | **Multivariate** | | |
|  | **r** | ***P*** |  | **Beta** | **Adjusted R^2^ change** | ***P*** |
| Age (years) | -0.019 | 0.853 |  |  |  |  |
| Height (cm) | 0.065 | 0.516 |  |  |  |  |
| Body weight (kg) | 0.304 | 0.002* |  |  |  |  |
| Waist circumference (cm) | 0.363 | < 0.001* |  |  |  |  |
| Body mass index (kg/m^2^) | 0.338 | 0.001* |  |  |  |  |
| Systolic blood pressure (mmHg) | 0.406 | < 0.001* |  | 0.293 | 0.098 | < 0.001* |
| Diastolic blood pressure (mmHg) | 0.193 | 0.053 |  |  |  |  |
| Pulse pressure (mmHg) | 0.315 | 0.001* |  |  |  |  |
| Total cholesterol (mg/dL) | 0.038 | 0.703 |  |  |  |  |
| Log-Triglycerides (mg/dL) | 0.319 | 0.001* |  |  |  |  |
| HDL-C (mg/dL) | -0.301 | 0.002* |  |  |  |  |
| LDL-C (mg/dL) | 0.083 | 0.409 |  |  |  |  |
| Log-Glucose (mg/dL) | 0.245 | 0.014* |  |  |  |  |
| Blood urea nitrogen (mg/dL) | 0.238 | 0.016* |  |  |  |  |
| Creatinine (mg/dL) | 0.281 | 0.004* |  |  |  |  |
| Glomerular filtration rate (mL/min) | -0.313 | 0.001* |  | -0.288 | 0.072 | 0.001* |
| Total calcium (mg/dL) | 0.168 | 0.093 |  |  |  |  |
| Phosphorus (mg/dL) | 0.052 | 0.607 |  |  |  |  |
| Calcium-phosphorous product (mg^2^/dL^2^) | 0.090 | 0.370 |  |  |  |  |
| Log-iPTH (pg/mL) | -0.157 | 0.118 |  |  |  |  |
| Log-CRP (mg/dL) | 0.416 | < 0.001* |  | 0.287 | 0.066 | 0.001* |
| Log-Insulin (uIU/mL) | 0.282 | 0.004* |  |  |  |  |
| Log-HOMA-IR | 0.323 | 0.001* |  |  |  |  |
| Log-HOMA2-IR | 0.307 | 0.002* |  |  |  |  |
| Log-Adiponectin (μg/mL) | -0.444 | < 0.001* |  | -0.268 | 0.189 | < 0.001* |

Data of triglyceride, glucose, iPTH, CRP, insulin, HOMA-IR, and adiponectin levels showed skewed distribution, and therefore were log-transformed before analysis.

Analysis data was done using the univariate linear regression analyses or multivariate stepwise linear regression analysis (adopted factors: diabetes, body weight, waist circumference, body mass index, systolic blood pressure, pulse pressure, log-triglyceride, HDL-C, log-glucose, blood urea nitrogen, creatinine, glomerular filtration rate, log-CRP, log-insulin, log-HOMA-IR, log-HOMA2-IR,and log-adiponectin).

HDL-C, high-density lipoprotein cholesterol; LDL-C, low-density lipoprotein cholesterol; iPTH, intact parathyroid hormone; CRP, C-reactive protein; HOMA-IR, homeostasis model assessment of insulin resistance.

Table S2. Correlation between right brachial-ankle pulse wave velocity levels and clinical variables among 101 hemodialysis patients

| **Variables** | **Right brachial-ankle pulse wave velocity (m/s)** | | | | | |
| --- | --- | --- | --- | --- | --- | --- |
|  | **Univariate** | |  | **Multivariate** | | |
|  | **r** | ***P*** |  | **Beta** | **Adjusted R^2^ change** | ***P*** |
| Age (years) | -0.061 | 0.544 |  |  |  |  |
| Height (cm) | 0.082 | 0.414 |  |  |  |  |
| Body weight (kg) | 0.385 | < 0.001* |  |  |  |  |
| Waist circumference (cm) | 0.451 | < 0.001* |  |  |  |  |
| Body mass index (kg/m^2^) | 0.425 | < 0.001* |  |  |  |  |
| Systolic blood pressure (mmHg) | 0.416 | < 0.001* |  | 0.299 | 0.119 | < 0.001* |
| Diastolic blood pressure (mmHg) | 0.239 | 0.016* |  |  |  |  |
| Pulse pressure (mmHg) | 0.294 | 0.003* |  |  |  |  |
| Total cholesterol (mg/dL) | 0.104 | 0.300 |  |  |  |  |
| Log-Triglycerides (mg/dL) | 0.389 | < 0.001* |  |  |  |  |
| HDL-C (mg/dL) | -0.292 | 0.003* |  |  |  |  |
| LDL-C (mg/dL) | 0.091 | 0.365 |  |  |  |  |
| Log-Glucose (mg/dL) | 0.300 | 0.002* |  |  |  |  |
| Blood urea nitrogen (mg/dL) | 0.267 | 0.007* |  |  |  |  |
| Creatinine (mg/dL) | 0.264 | 0.008* |  |  |  |  |
| Glomerular filtration rate (mL/min) | -0.301 | 0.002* |  | -0.237 | 0.040 | 0.003* |
| Total calcium (mg/dL) | 0.176 | 0.079 |  |  |  |  |
| Phosphorus (mg/dL) | 0.074 | 0.464 |  |  |  |  |
| Calcium-phosphorous product (mg^2^/dL^2^) | 0.113 | 0.262 |  |  |  |  |
| Log-iPTH (pg/mL) | -0.173 | 0.083 |  |  |  |  |
| Log-CRP (mg/dL) | 0.433 | < 0.001* |  | 0.264 | 0.067 | 0.002* |
| Log-Insulin (uIU/mL) | 0.325 | 0.001* |  |  |  |  |
| Log-HOMA-IR | 0.381 | < 0.001* |  |  |  |  |
| Log-HOMA2-IR | 0.359 | < 0.001* |  |  |  |  |
| Log-Adiponectin (μg/mL) | -0.446 | < 0.001* |  | -0.209 | 0.027 | 0.018* |

Data of triglyceride, glucose, iPTH, CRP, insulin, HOMA-IR, and adiponectin levels showed skewed distribution, and therefore were log-transformed before analysis.

Analysis data was done using the univariate linear regression analyses or multivariate stepwise linear regression analysis (adopted factors: diabetes, body weight, waist circumference, body mass index, systolic blood pressure, diastolic blood pressure, pulse pressure, log-triglyceride, HDL-C, log-glucose, blood urea nitrogen, creatinine, glomerular filtration rate, log-CRP, log-insulin, log-HOMA-IR, and log-adiponectin).

HDL-C, high-density lipoprotein cholesterol; LDL-C, low-density lipoprotein cholesterol; iPTH, intact parathyroid hormone; CRP, C-reactive protein; HOMA-IR, homeostasis model assessment of insulin resistance.
